# Linking Virus Genomes with Host Taxonomy

**DOI:** 10.3390/v8030066

**Published:** 2016-03-01

**Authors:** Tomoko Mihara, Yosuke Nishimura, Yugo Shimizu, Hiroki Nishiyama, Genki Yoshikawa, Hideya Uehara, Pascal Hingamp, Susumu Goto, Hiroyuki Ogata

**Affiliations:** 1 Institute for Chemical Research, Kyoto University, Uji, Kyoto 611-0011, Japan; mihara@kuicr.kyoto-u.ac.jp (T.M.); yosuke@kuicr.kyoto-u.ac.jp (Y.N.); shimizu@kuicr.kyoto-u.ac.jp (Y.S.); hiroki@kuicr.kyoto-u.ac.jp (H.N.); yos@kuicr.kyoto-u.ac.jp (G.Y.); pascal.hingamp@univ-amu.fr (P.H.); goto@kuicr.kyoto-u.ac.jp (S.G.); 2 SGI Japan, Ltd., Yebisu Garden Place Tower 31F, 4-20-3 Ebisu Shibuya-ku, Tokyo 150-6031, Japan; uehara@sgi.com; 3 Aix Marseille Université, CNRS, IGS UMR 7256, 13288 Marseille, France

**Keywords:** virus-host interactions, database, taxonomy, GenomeNet, KEGG, genomes

## Abstract

Environmental genomics can describe all forms of organisms—cellular and viral—present in a community. The analysis of such eco-systems biology data relies heavily on reference databases, e.g., taxonomy or gene function databases. Reference databases of symbiosis *sensu lato*, although essential for the analysis of organism interaction networks, are lacking. By mining existing databases and literature, we here provide a comprehensive and manually curated database of taxonomic links between viruses and their cellular hosts.

## 1. Introduction

Viruses are found in all three domains of life, from higher animals to tiny prokaryotes [1], and some viruses even infect other viruses (*i.e.*, virophages) [2]. Given the rapid increase of sequenced viruses infecting diverse hosts, it is becoming increasingly possible to study viruses from the wider taxonomic perspective of global interaction networks of both viruses and hosts. The first complete genome ever sequenced was in fact a viral genome, that of bacteriophage ФX174 (5375 nucleotide circular ssDNA, infecting *Escherichia coli)* determined in 1977 by Frederick Sanger and his colleagues [3]. Currently, 6544 complete viral genomes are recorded in the National Center for Biotechnology Information (NCBI) Reference Sequence Database (RefSeq release 72) [4]. Given this large number of viral genomes, comparative genomics is a potent approach to studying viruses with the aim of uncovering shared functional features, deducing ancient evolutionary histories, or predicting host ranges for known and novel viruses.

Host information is an absolutely essential component in such approaches, since viral replication is dependent on host organisms. For instance, accessing the genomic and taxonomic information of both viruses and their hosts is a prerequisite to investigate nucleotide/codon composition correlations in viral and host genomes [5], to reveal co-evolution [6,7], and to detect genetic interactions by horizontal gene transfers between viruses and their hosts [8]. However, RefSeq stores host information in the form of free text corresponding to host species names (e.g., *Homo sapiens*, *Sus scrofa*, *etc.*), host names (e.g., human, pig, *etc.*), or host-related adjectives (e.g., human, porcine, *etc.*) for 67% of the viral genome entries, with no specification of the widely used NCBI taxonomic identifiers (TaxIDs). Being typical of free text annotation fields, these host names suffer from many cases of ambiguities or typos that make reliable automated mapping to TaxIDs difficult. For example, “*Sida* sp.” is provided as the host information for *Sida mosaic Sinaloa virus* (NC_008059) in RefSeq, but “*Sida*” could refer not only to a genus of angiosperms (the true host group, TaxID = 108335) but also to a genus of crustaceans (non-host, TaxID = 77655). UniProtKB [9] does provide machine readable TaxIDs for viruses and hosts, but this linking information is only assigned for 20% of viral genomes in RefSeq. To our knowledge, there is no comprehensive resource that organizes robust machine readable taxonomic links between viruses and their hosts. Consequently, even such a simple query as “How many archaeal virus genomes are recorded in RefSeq?” is not easy to answer for non-specialists, and is completely out of reach for automated software methods.

## 2. Materials and Methods

The GenomeNet Virus-Host Database [10] organizes TaxID based links between viruses and their hosts. We first extracted *natural host* and *laboratory host* information from RefSeq viral genome entries (“source” features) and from protein sequence entries in UniProtKB (“OX—organism taxonomy cross-reference” and “OH—organism host” entry lines). RefSeq free text to TaxID mapping was manually curated (error correction and disambiguation), and for viral genome entries that lacked relevant host data, we collected host information by surveying the literature. In some cases, we referred to viral species names (or virus names) to reach host information as some of these names contain host names (e.g., *Apple mosaic virus*). Finally, the Virus-Host Database provides links to external reference resources such as ViralZone [11], the NCBI taxonomy database, the Kyoto Encyclopedia of Genes and Genomes database [12], and the International Committee on Taxonomy of Viruses database [13].

## 3. Results and Discussion

**Figure 1:**
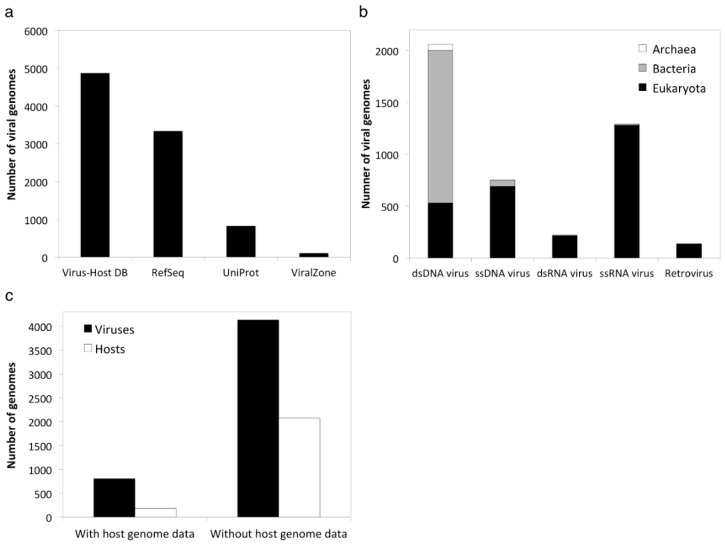
GenomeNet Virus-Host Database. (**a**) Comparison of the number of viral genomes with host information in different databases; (**b**) Number of viral genomes in the Virus-Host Database across different groups of viruses with information of host taxonomic domain; (**c**) Number of viruses in the Virus-Host Database with or without links to host genomic sequence data.

Currently, about 38% of the total viral entries in the Virus-Host Database are manually curated. These curated entries can be distinguished from automatically created entries by the “Evidence” line in the individual viral entry page of the database. Manually curated entries contain “Literature” and/or “Other” keyword depending on the type of evidence for known virus-host relationships, whereas automatically created entries contain “RefSeq” and/or “UniProt”. The number of viral genomes, host taxonomy and host genome sequence availability are summarized in Figure 1. To illustrate how the paired taxonomic information could be used in computational genome analysis, here we present two broad scale analyses spanning the full range of sequenced prokaryotic viruses with known hosts.

**Figure 2:**
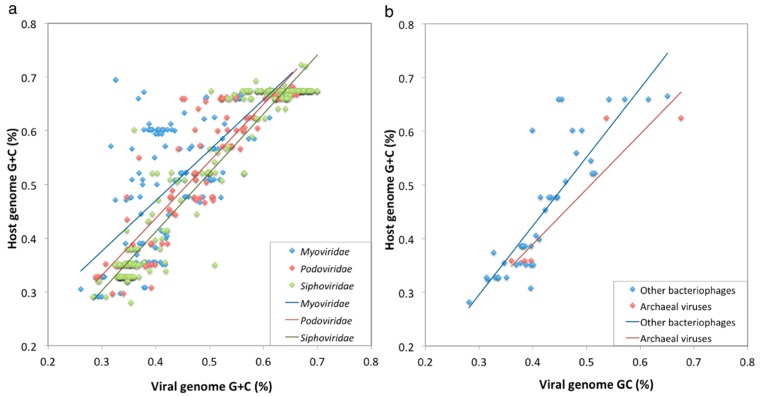
Viral and host genomic G + C content. Genomic G+C% for 746 virus-host genome pairs for *Caudovirales* (**a**) and 51 other prokaryotic viruses (**b**) are plotted. Pearson’s correlation coefficients are as follows: *Myoviridae*: *r* = 0.755, *p* = 2.73 × 10^−39^, *n* = 206; *Myoviridae* without tRNA genes: *r* = 0.945, *p* = 2.12 × 10^−32^, *n* = 65; *Myoviridae* with tRNA genes: *r* = 0.703, *p* = 2.67 × 10^−22^, *n* = 141; *Podoviridae*: *r* = 0.892, *p* = 1.63 × 10^−40^, *n* = 114; *Siphoviridae*: *r* = 0.969, *p* = 9.94 × 10^−261^, *n* = 426; Other bacteriophages: *r* = 0.864, *p* = 2.09 × 10^−14^, *n* = 45; Archaeal viruses: *r* = 0.931, *p* = 6.99 × 10^−3^, *n* = 6. Lines in the plot areas indicate linear regressions by the least squares method.

First, we examined the genomic G+C% (Figure 2). The results showed significant correlations in the genomic G+C% between viruses and their hosts across different groups of viruses, being consistent with previous observations [14]. Since host organisms provide a variety of molecular building blocks and machinery required for viral reproduction, the nucleotide compositions of viruses can reflect the adaptation to their surrounding cellular environments or to host machinery. The correlation was weaker for *Myoviridae* (*r* = 0.755) than for other *Caudovirales* (*Siphoviridae*, *r* = 0.969; *Podoviridae*, *r* = 0.892) (Figure 2a), which could be explained by the existence of broad host range myoviruses encoding tRNA genes [15]. Indeed, when we analyzed myoviruses without tRNA genes, we obtained a higher correlation coefficient (*r* = 0.945).

**Figure 3:**
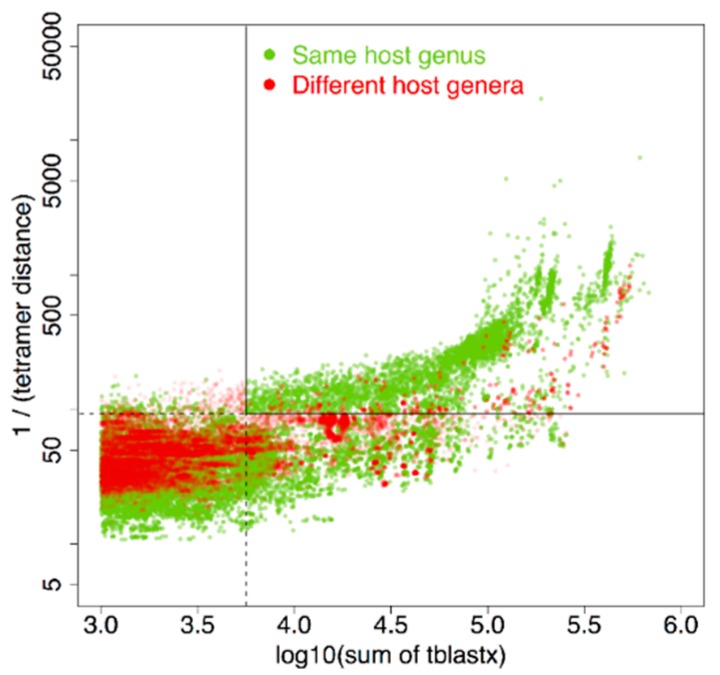
Assessment of the host range predictability based on viral genomic similarities. Dot plot of virus genomic similarity estimated by two measures: tetramer similarity (*y* axis) and protein alignment scores (*x* axis). Each dot represents a pair of virus genomes. The vertical (*x* = 3.75) and horizontal (*y* = 93) lines are the thresholds delineating the top right sector corresponding to same host genus prediction with a false discovery rate of 4.58%. The colors of the dots indicate if the two viruses have the same host (green) or not (red).

Second, we used the paired taxonomic information to assess a computational method for host prediction. Genomic features of phages and their hosts can be used to predict phage-host pairs [16,17]. Here, we analyzed the relationship between the host taxonomic similarity and phage inter-genome sequence similarity. The underlying assumption is that if two phages have similar enough genomes in terms of both sequence and nucleotide composition similarities, then the two phages may be evolutionarily highly related and thus may share the same or related hosts. From the Virus-Host Database, we extracted a set of 1,057 genomes of phages that are known to infect 107 distinct bacteria. As a proxy for overall phage genome sequence similarity, we used the logarithm of the sum of the TBLASTX scores [18] between the two phage genomes. For compositional similarity, we used one divided by the Euclidean distance [19] based on tetramer frequencies. As shown in Figure 3, phages showing high inter-genomic similarity by both measures tend to infect similar hosts (*i.e.*, same host taxonomy at genus level). By setting appropriate thresholds for sequence and compositional similarities (>3.75 and >93, respectively), we were able to predict pairs of phages infecting hosts of the same genus with a precision of 95.42% and a recall of 11.5%. This method solely relies on the genomic data of phages (*i.e.*, sequence and nucleotide composition similarities between phage genomes) and correctly predicted 4582 phage pairs with the same host genus (between 759 distinct phages). In comparison, Roux *et al.* [17] previously reported a prediction method based on nucleotide composition similarities between phage and host genomes with a higher precision (98.98%) but with a lower recall (0.76%) than our method. Therefore, the presented approach holds a potential (which will grow as reference databases are enriched) for predicting hosts of unknown phage sequences (such as those obtained by metagenomics projects [20]) solely based on their similarity with known viral genomes.

The GenomeNet Virus-Host Database provides machine readable taxonomic links between completely sequenced viral genomes and their hosts. The taxonomic links are extracted from existing databases and literature. The database is updated upon each new RefSeq release with its content being continuously improved/enriched by manual curation. We also welcome users to provide feedback on the functionality and contents of the database. The Virus-Host Database is accessible through a user friendly web interface and as a downloadable file.
